# Elastography Enhances Diagnostic Accuracy of ACR TI-RADS in Thyroid Nodule Evaluation

**DOI:** 10.1210/clinem/dgaf670

**Published:** 2025-12-18

**Authors:** Nikolaos Angelopoulos, Dimitrios G Goulis, Ioannis Chrisogonidis, Ioannis Iakovou, Franklin N Tessler

**Affiliations:** 2nd Academic Department of Nuclear Medicine, AHEPA University Hospital, Faculty of Medicine, School of Health Sciences, Thessaloniki 54636, Greece; Unit of Reproductive Endocrinology, 1st Department of Obstetrics and Gynecology, Faculty of Medicine, School of Health Sciences, Aristotle University of Thessaloniki, Thessaloniki 54636, Greece; Department of Radiology, Faculty of Medicine, School of Health Sciences, Aristotle University of Thessaloniki, Thessaloniki 54636, Greece; 2nd Academic Department of Nuclear Medicine, AHEPA University Hospital, Faculty of Medicine, School of Health Sciences, Thessaloniki 54636, Greece; Department of Radiology, University of Alabama at Birmingham, Birmingham, AL 35249, USA

**Keywords:** thyroid nodule, FNA, ACR TI-RADS, elastography, ultrasonography

## Abstract

**Context:**

The American College of Radiology Thyroid Imaging Reporting and Data System (ACR TI-RADS) incorporates conventional grayscale ultrasonography (US) as the only imaging technique without considering the clinical and demographic characteristics of patients.

**Objective:**

This study assessed whether the addition of demographic information, color Doppler US (CDUS), and strain elastography (SE) could enhance malignancy risk stratification beyond the current ACR TI-RADS criteria.

**Methods:**

This prospective study enrolled 556 adult patients with thyroid nodules 10 mm or greater who were referred for fine-needle aspiration (FNA) according to the ACR TI-RADS recommendations. All nodules underwent standardized US evaluations and vascularity assessments using CDUS and SE, with cytological analysis performed according to the Bethesda system. Surgical pathology was the gold standard for malignancy when available.

**Results:**

Applying elastography ratio (ER) thresholds (>1.60, >0.44, and >0.54 for ACR TI-RADS categories 3, 4, and 5, respectively) as an additional criterion for FNA reduced the number of procedures from 501 to 260, without missing any malignant cases. Notably, elastography demonstrated an excellent discriminative performance in ACR TI-RADS 3 nodules (Youden index 0.994, area under the curve 0.994), supporting its value in improving risk stratification in this challenging, predominantly benign category.

**Conclusion:**

Integrating elastography into the ACR TI-RADS framework can optimize FNA utilization in the management of thyroid nodules by reducing the number of unnecessary aspiration biopsies.

The Thyroid Imaging Reporting and Data System (TIRADS) was established in 2009 as a structured tool to estimate the likelihood of malignancy in thyroid nodules by analyzing specific ultrasonography (US) patterns (1). Since then, additional risk stratification systems (RSS), such as the European TIRADS (EU-TIRADS), American College of Radiology TIRADS (ACR TI-RADS) (2, 3), and K-TIRADS (4) have been developed. All RSS are based on 2-dimensional (B-mode) US characteristics, which do not account for clinical or other imaging features such as color Doppler sonography (CDUS) and elastography. CDUS, along with more advanced techniques such as microvascular imaging, is designed to depict vascularity within thyroid nodules. While the clinical utility of vascular patterns in distinguishing benign from malignant nodules remains a subject of ongoing investigation (5), malignant nodules often present with increased central blood flow, a feature that is less common in benign lesions (6).

While the importance of clinical and demographic characteristics is well recognized, these factors have not been formally incorporated into the RSS when estimating a nodule's risk of malignancy or recommending management. There is growing interest in the influence of regional variations in the prevalence of malignancies, as this may be shaped by several confounding elements, such as sex, radiation exposure, and genetic predisposition (7, 8).

Real-time elastography (RTE) and strain elastography (SE) (9) have been used to characterize thyroid nodules more accurately. These techniques assess the mechanical stiffness of tissues, with lower levels suggesting benignity, as benign tissues tend to be softer and less rigid (10-12). However, elastography has not been widely adopted, largely because of the diversity of available techniques and the absence of standardized protocols.

The wider application of RSS has reduced the number of unnecessary fine-needle aspirations (FNAs). Nevertheless, some benign nodules continue to undergo aspirations (13). Therefore, the present study aimed to investigate whether incorporating demographic, clinical, and additional US characteristics in patients who meet the criteria for FNA according to the ACR TI-RADS could further improve its diagnostic accuracy.

## Materials and Methods

### Study Population

This study was conducted in accordance with the ethical principles of the Declaration of Helsinki. The study was approved by the institutional review board of the Aristotle University of Thessaloniki (Bioethics Committee; protocol No. 93/2024). All participants provided written informed consent prior to enrollment, in accordance with the requirements of the institutional review board.

Consecutive adult individuals aged 18 years or older, presenting with at least 1 thyroid nodule 10 mm or greater in maximum diameter, were prospectively enrolled between May 2023 and April 2025 at the Endocrinology and Nuclear Medicine Departments of AHEPA University Hospital in Thessaloniki, Greece. Thyroid US was performed for various clinical indications, including palpable nodules identified on physical examination, incidentally discovered nodules on other imaging studies (such as computed tomography, magnetic resonance imaging, or carotid Doppler), and the evaluation or follow-up of patients with known or suspected autoimmune thyroiditis (eg, Hashimoto disease). The population in the region where the study was conducted had sufficient iodine intake (14). Patients with a personal history of thyroid cancer or previously biopsied nodules and those who declined to undergo FNA were excluded.

All participants underwent comprehensive clinical evaluation prior to imaging. This evaluation included the collection of demographic data (age and sex), relevant medical and family history (eg, thyroid disease and autoimmune conditions), and baseline laboratory assessments as part of the standard diagnostic work-up. Laboratory measurements included thyrotropin, free thyroxine (fT_4_), antithyroid peroxidase antibodies, and antithyroglobulin antibodies.

Autoimmune thyroiditis was diagnosed based on a documented history of elevated antithyroid peroxidase antibodies and/or antithyroglobulin antibody titers, irrespective of antibody status at the time of study participation. Basal serum calcitonin concentrations were used to screen for medullary thyroid carcinoma, and individuals with elevated calcitonin concentrations were excluded from the study. In cases of suppressed thyrotropin concentrations, thyroid scintigraphy was performed to detect autonomously functioning thyroid nodules, which constituted part of the exclusion criteria. Thyroid US was performed on all patients using a standardized protocol, as described later. Clinical data were prospectively collected using a standardized questionnaire completed at the time of evaluation. Cases with missing data were excluded from the relevant analyses.

### Ultrasound Procedures

Thyroid US was performed using a LOGIQ P9 system (General Electric Medical Systems) with an 8- to 12-MHz multifrequency linear transducer. All US examinations were conducted and interpreted by a single operator (N.A.) with more than 20 years of experience in thyroid imaging. Each nodule was assessed using B-mode US, RTE, and CDUS, as detailed later.

The number of thyroid nodules was recorded during the US examination, and a comprehensive assessment of their characteristics was performed based on the ACR TI-RADS criteria (3). The documented features included the dimensions of each nodule in 3 planes (anteroposterior, transverse, and longitudinal); shape (wider-than-tall or taller-than-wide); composition (solid, cystic, spongiform, or mixed); echogenicity (hyperechoic, isoechoic, hypoechoic, or very hypoechoic); margin (smooth, ill-defined, lobulated/irregular, or presence of extrathyroidal extension); and echogenic foci (comet-tail artifacts, peripheral calcifications, macrocalcifications, or punctate echogenic foci).

Blood flow patterns within the nodules were assessed using CDUS on the same system. The Doppler settings were optimized for thyroid imaging, with a low pulse repetition frequency (typically 700-1000 Hz), low wall filter, and high color gain just below the noise threshold to enhance the sensitivity for detecting low-velocity intranodular and perinodular blood flow. The nodules were categorized as A (peripheral blood flow), B (central blood flow), C (mixed [central and peripheral] blood flow), or D (no blood flow).

SE was performed using the same US device for all thyroid nodules. Gentle external compression was applied to obtain valid elastographic images. Interpretation was based on a color-coded elastographic map, where red corresponds to soft tissue, green to intermediate stiffness (ie, equal strain), and blue to rigid tissues exhibiting little to no deformation.

A semi-quantitative evaluation was performed by calculating the strain ratio (SR), also referred to as the elastography ratio (ER). This numerical index represents the ratio between 2 regions of interest located at similar depths: the mean strain value of the nodule (E2) and the strain value of the adjacent sternocleidomastoid muscle (E1), such that SR = E2/E1. Transverse plane imaging was used to ensure that the nodule and reference muscle were included within the same imaging frame, thereby enhancing the accuracy and reproducibility of the measurements.

Each thyroid nodule was classified according to the ACR TI-RADS by an experienced endocrinologist with more than 20 years of experience in thyroid US. The classifications were performed prospectively during US examinations and were subsequently used for analysis. Accordingly, when indicated, thyroid nodules were subjected to FNA based on their ACR TI-RADS category and maximum nodule diameter. FNAs were performed at a single specialized radiology center with expertise in thyroid FNA procedures within 1 month of the diagnostic US. All FNA procedures were performed under real-time ultrasound guidance using 25- to 27-gauge needles in accordance with standard cytological practice. For each nodule, 3 to 4 needle passes were performed to ensure sample adequacy and diagnostic accuracy.

Cytological findings obtained from FNA were interpreted using the Bethesda System for Reporting Thyroid Cytopathology (15). FNA was repeated for patients with Bethesda I and III results. Based on the FNA cytology results, thyroidectomy was recommended for patients whose nodules were classified as Bethesda categories III (AUS/FLUS), IV (FN/SFN), V (suspicious for malignancy), or VI (malignant). The corresponding histopathological diagnoses were recorded and analyzed in cases where nodules were surgically removed. Histopathological examinations were performed in 2 tertiary referral centers with substantial experience in thyroid cancer pathology: 1 oncology hospital specializing in head and neck malignancies, and another high-volume endocrine surgery institution where numerous thyroidectomies are performed annually. This dual-center approach ensured high diagnostic accuracy and consistency of histopathological evaluation. In contrast, Bethesda II nodules were managed conservatively without surgery, in strict adherence to the current TIRADS-based recommendations. Therefore, the reference standard was histopathology for surgically resected nodules and cytology for nodules classified as Bethesda II. These recommendations followed institutional clinical guidelines and were made in the absence of genetic or molecular testing, which was unavailable.

### Statistical Analysis

The Kolmogorov-Smirnov test was applied to evaluate the distribution of continuous variables and to assess whether they followed a normal (gaussian) distribution. For variables that did not demonstrate normality, data are expressed as median values accompanied by the interquartile range. Categorical variables were described using absolute frequency and percentage.

Backward logistic regression analysis was employed to evaluate the potential predictive value of the variables under investigation for thyroid malignancy. This analysis was performed in the overall cohort of patients who underwent FNA and within each ACR TI-RADS category. Receiver operating characteristic (ROC) curve analyses were conducted to evaluate the ability of SR to distinguish between benign and malignant thyroid nodules. The ROC methodology was further employed to identify optimal threshold values and calculate the corresponding sensitivity, specificity, positive predictive value, negative predictive value, and area under the ROC curve (AUC), thereby assessing the overall diagnostic performance of the SR. Finally, the optimal threshold values of SR in each ACR TI-RADS category were determined to identify the thresholds with the highest sensitivity for ruling out thyroid cancer. All statistical analyses were performed using MedCalc version 19.5.3 (MedCalc Software). Statistical significance was set at a *P* value less than or equal to .05.

## Results

### Total Population

A total of 3150 patients were examined, and although all thyroid nodules were prospectively evaluated during US examination, only the largest nodule per patient was included in the analysis to ensure methodological consistency. Specifically, in cases where multiple nodules were present, the nodule selected for inclusion and/or FNA was the largest nodule that met the ACR TI-RADS criteria for aspiration, as determined by its risk category (TR score) and maximum diameter. This strategy was adopted to align with guideline-based clinical practice, ensure methodological uniformity, and avoid statistical bias related to the clustering of multiple nodules within the same patient. However, it is acknowledged that the largest nodule is not necessarily the one with the highest risk score. Nodules smaller than 1 cm (n = 1260) were excluded from the analysis in accordance with the ACR TI-RADS guidelines, which generally do not recommend FNA for subcentimeter nodules unless specific high-risk features are present. Therefore, this exclusion criterion was applied as part of the study design to align with standard clinical practice. Of the remaining 1890 nodules, 459 classified into categories 1 and 2 were excluded from the study as further evaluation with FNA was not recommended. Ultimately, 1431 nodules were included in this analysis. According to the ACR TI-RADS stratification, 574 patients (40.1%) met the criteria and were advised to undergo FNA cytology of thyroid nodules (Table 1).

**Table 1.** Fine-needle aspiration indication according to the American College of Radiology Thyroid Imaging Reporting and Data System

ACR TI-RADS category	Nodules (n)	FNA indication (n) (%)
3	747	169 (22.6%)
4	630	351 (55.7%)
5	54	54 (100%)
Total	1431	574 (40.1%)

Abbreviations: ACR TI-RADS, American College of Radiology Thyroid Imaging Reporting and Data System; FNA, fine-needle aspiration.

**Figure 1.**
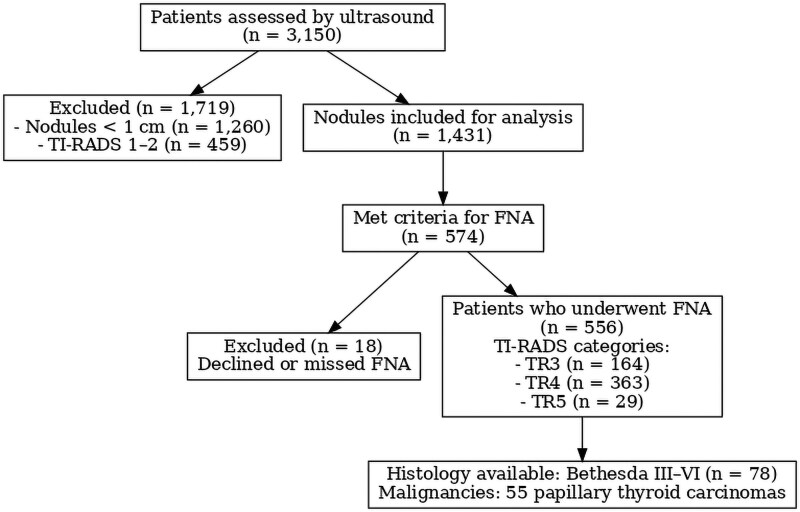
Study flow diagram. Flowchart summarizing the recruitment and selection of patients undergoing thyroid ultrasound and strain elastography. ACR TI-RADS, American College of Radiology Thyroid Imaging Reporting and Data System; FNA, fine-needle aspiration.

Eighteen patients (11 women, 7 men) either declined to undergo FNA or failed to attend their scheduled appointments and were excluded from further analysis. Finally, data from 556 patients (451 women [81.1%] and 105 men [18.9%]) who underwent FNA were analyzed. Fig. 1 illustrates the patient flow of the study. The study population had a median age of 58 years (range, 18-90 years), median body mass index (BMI) of 26.4 (range, 15.8-54.8), and median nodule diameter of 23 mm (range, 10-47 mm). BMI and nodule size did not follow a normal distribution, as confirmed by Kolmogorov-Smirnov and Shapiro-Wilk tests.

Of these, 27.9% were diagnosed with autoimmune thyroiditis, and 28.8% had hypothyroidism and were receiving T4 replacement therapy (Supplementary Table S1) (16).

The cytological results are shown in Table 2. All patients with indeterminate cytological diagnoses (Bethesda categories III and IV, n = 22 and n = 13, respectively) or with cytology suspicious for malignancy (Bethesda category V, n = 25) and those with malignant cytology (Bethesda category VI, n = 18) underwent surgical treatment. Histopathological examination revealed 55 papillary thyroid cancers, either classic variant or follicular variant (Table 3). Supplementary Table S2 (16) summarizes the cases described as discordant between cytology and histology. Most fall within the expected diagnostic spectrum of the Bethesda III to V and TR 3 to 5 categories. The main discordance involved papillary thyroid carcinomas classified as Bethesda IV, largely corresponding to follicular variants, which explains their cytological overlap with follicular-patterned lesions. The descriptive characteristics of the TI-RADS categories are shown in Supplementary Table S3 (16). The mean BMI did not significantly differ between patients with benign and malignant nodules (27.5 ± 4.6 vs 27.8 ± 4.4; *P* = .42) and showed no consistent trend across ACR TI-RADS categories.

**Table 2.** Fine-needle aspiration results (Bethesda category) according to the American College of Radiology Thyroid Imaging Reporting and Data System

ACR TI-RADS category	Bethesda category					
	II	III	IV	V	VI	Total
3	156	4	3	1	0	164
4	297	15	7	11	13	343
5	25	3	3	13	5	49
Total	478	22	13	25	18	556

Abbreviations: ACR TI-RADS, American College of Radiology Thyroid Imaging Reporting and Data System; FNA, fine-needle aspiration.

**Table 3.** Histological findings from thyroidectomy specimens according to the Bethesda category and American College of Radiology Thyroid Imaging Reporting and Data System

ACR TI-RADS category	Bethesda category											
	II		III		IV		V		VI		Total	
	FNA	Malignancy	FNA	Malignancy	FNA	Malignancy	FNA	Malignancy	FNA	Malignancy	FNA	Malignancy
3	156	N/A	4	1	3	2	1	1	0	0	164	4
4	297	N/A	15	3	7	4	11	11	13	13	343	31
5	25	N/A	3	1	3	2	13	12	5	5	49	20
Total	478	N/A	22	5	13	8	25	24	18	18	556	55

Abbreviations: ACR TI-RADS, American College of Radiology Thyroid Imaging Reporting and Data System; FNA, fine-needle aspiration; N/A, nonapplicable.

**Figure 2.**
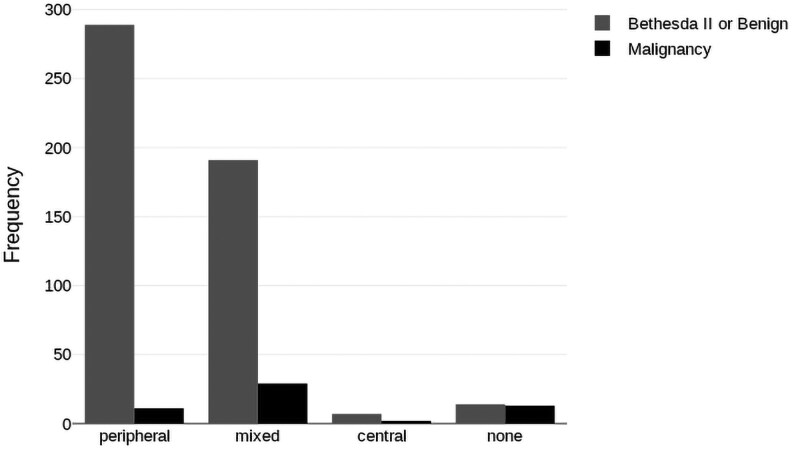
Malignancy rate according to ultrasonographic vascularity pattern. Bar graph demonstrating the proportion of malignant nodules among the 556 cases subjected to fine-needle aspiration, stratified by color Doppler vascularity pattern (peripheral, mixed, absent, central).

Among the evaluated nodules, the majority demonstrated peripheral vascularization (54.0%), followed by mixed (39.6%), absent (4.9%), and central (1.6%) blood flow patterns (Table 4). Malignancy was most frequently observed in nodules with mixed or absent vascularization, whereas nodules with peripheral vascularity were predominantly benign. A statistically significant association was found between vascularity pattern and malignancy (χ² = 61.58; *P* < .001; see Table 4 and Fig. 2). The elastographic assessment yielded a median E2/E1 stiffness ratio of 0.6, with an interquartile range of 0.5, a minimum value of 0.1, and a maximum value of 12.5. The vascularity and clinical features according to the TI-RADS category are shown in Supplementary Table S4 (16).

**Table 4.** Malignant nodules evaluated by fine-needle aspiration and classified according to vascularity pattern on color Doppler imaging

Vascularity pattern	n	%	Malignancy			
			n	% of total	% in category	% Malignant
Peripheral	300	54.0	11	2.0	3.7	20.0
Mixed	220	39.6	29	5.2	13.2	52.7
None	27	4.9	13	2.3	48.2	23.6
Central	9	1.6	2	0.4	22.2	3.6
Total	556	100	55	9.9		100

Abbreviation: FNA, fine-needle aspiration.

Further statistical analyses were conducted to comprehensively evaluate all potential demographic, clinical, and US factors that could predict the presence of malignancy. Α multivariate model was developed that included sex, age, BMI, presence of autoimmune thyroiditis, presence of hypothyroidism, nodule size, vascularity pattern, and ER. Other historical factors known to be associated with thyroid malignancy were not considered because the ACR TI-RADS is not intended for high-risk individuals. Backward logistic regression analysis identified statistical significance for nodule size, presence of thyroiditis, absence of vascularity, mixed vascularity, and ER. The model demonstrated overall statistical significance (*P* < .001) in predicting thyroid malignancy (Table 5).

**Table 5.** Logistic regression coefficients and standard errors (total population)

Variable	Coefficient	SD	Wald	OR	95% CI	*P*
Size, mm	−0.08	0.03	5.77	0.92	0.86-0.98	.016
AT	0.93	0.40	5.51	2.54	1.16-5.55	.018
ER	1.71	0.23	53.46	5.52	3.49-8.72	<.001
No vascularity	2.23	0.64	12.05	9.33	2.64-32.92	<.001
Mixed vascularity	1.06	0.42	6.46	2.87	1.27-6.48	.011
Constant	−3.59	0.86	17.47			<.001

Abbreviations: AT, autoimmune thyroiditis; ER, elastography ratio; OR, odds ratio.

**Figure 3.**
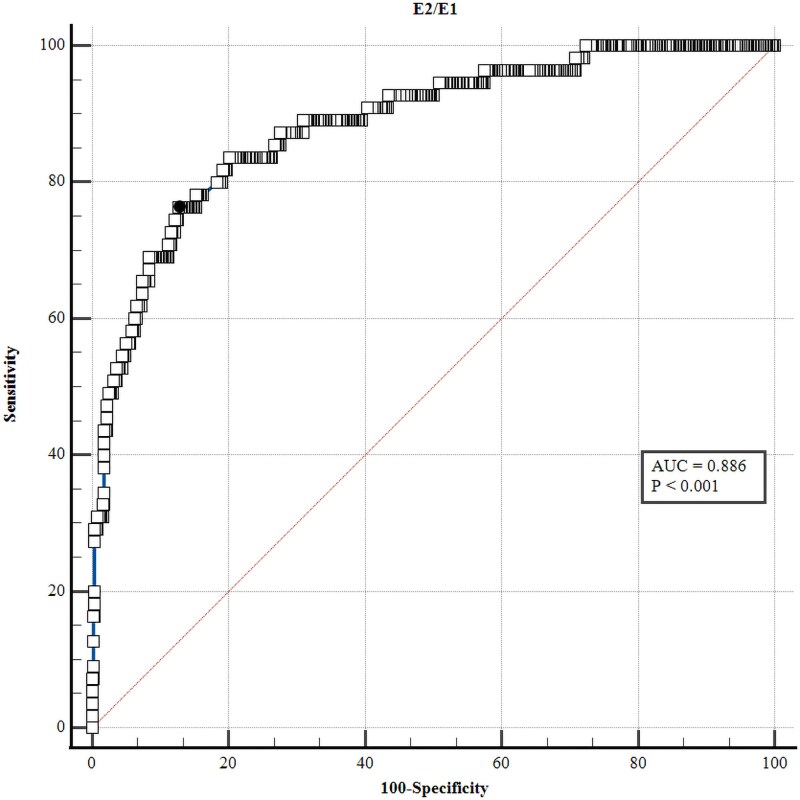
Receiver operating characteristic (ROC) curve for strain elastography (E2/E1 ratio [ER]) in differentiating benign from malignant thyroid nodules. ROC curve evaluating the diagnostic performance of the ER (E2/E1) in predicting malignancy. The optimal cutoff derived was ER greater than 1.13, corresponding to a sensitivity of 76.4% and specificity of 87.2%. AUC, area under the curve; FNA, fine-needle aspiration.

An ROC curve was generated to evaluate the discriminative ability of the E2/E1 muscle ratio. The AUC was 0.886, with a Youden index of 0.636 and an associated criterion of ER greater than 1.13 (sensitivity 76.4%, specificity 87.2%), as shown in Fig. 3.

### Diagnostic Performance of Elastography Across American College of Radiology Thyroid Imaging Reporting and Data System Categories

To delineate the independent diagnostic contribution of RTE, a stratified analysis was conducted across ACR TI-RADS categories, focusing exclusively on the ER metrics. ROC analyses were performed to identify the optimal ER thresholds for malignancy prediction within each subgroup (Table 6) and to avoid FNA procedures for each TIRADS category (Table 7).

- **ACR TI-RADS 3.** The ER demonstrated exceptional discriminatory capacity, with an AUC of 0.994 and a corresponding Youden index of 0.994. The optimal threshold was identified as ER greater than 1.60 (95% CI, 1.4-1.6), achieving 100% sensitivity and 99.4% specificity. The application of this threshold could have potentially obviated 159 FNA procedures, accounting for 96.9% of FNAs otherwise indicated solely based on the ACR TI-RADS criteria (Supplementary Fig. S1) (16).
- **ACR TI-RADS 4.** The ER retained robust diagnostic utility, yielding an AUC of 0.897 and a Youden index of 0.694. The derived threshold of ER greater than 1.37 (95% CI, 1.095-2.153) provided a sensitivity of 80.7% and specificity of 88.8%. The adoption of this threshold would have precluded 276 FNAs, representing 88.5% of those recommended by the ACR TI-RADS criteria alone (Supplementary Fig. S2) (16).
- **ACR TI-RADS 5.** Although the diagnostic performance was comparatively attenuated, the ER still yielded acceptable accuracy, with an AUC of 0.812 and a Youden index of 0.528. The optimal threshold was calculated at ER greater than 0.95 (95% CI, 0.633-1.812), corresponding to 70.0% sensitivity and 82.8% specificity. The implementation of this threshold would have eliminated 23 unnecessary FNAs (79.3%) (Supplementary Fig. S3) (16).

**Table 6.** Predictive value of elastography ratio from logistic regression

ACR TI-RADS category	ER coefficient	SE	*P*	OR	95% CI
3	4.53	1.42	.001	92.32	5.70-1494
4	1.67	0.26	<.001	5.29	3.17-8.84
5	3.0	1.08	.005	20.03	2.43-165

Abbreviations: ACR TI-RADS, American College of Radiology Thyroid Imaging Reporting and Data System; ER, elastography ratio; OR, odds ratio.

**Table 7.** Diagnostic performance of elastography ratio per American College of Radiology Thyroid Imaging Reporting and Data System category

ACR TI-RADS category	Total nodules	Malignant	Benign	ER threshold	TP	FP	FN	TN	Sensitivity, %	Specificity, %	FNAs avoided, n	FNAs avoided, %
3	164	4	160	>1.60	4	1	0	159	100.0	99.4	159	96.9
4	343	31	312	>1.37	25	36	6	276	80.7	88.8	276	88.5
5	49	20	29	>0.95	14	6	6	23	70.0	82.8	23	79.3

Abbreviations: ACR TI-RADS, American College of Radiology Thyroid Imaging Reporting and Data System; ER, elastography ratio; FN, false negative; FNA, fine-needle aspiration; FP, false positive; TN, true negative; TP, true positive.

To further enhance the sensitivity, alternative lower thresholds were explored. For ACR TI-RADS 4, an ER greater than 0.44 allowed the safe omission of 71 of 312 FNAs (22.8%) without overlooking any malignant lesions. Similarly, in the ACR TI-RADS 5 cohort, an ER greater than 0.54 excluded 11 of 29 FNAs (38.0%) with no false negatives. Overall, the integration of SE-derived ER thresholds into the existing ACR TI-RADS framework decreased the number of FNAs from 501 to 260 (48%) without compromising the sensitivity for thyroid cancer detection.

## Discussion

Although EU-TIRADS and ACR TI-RADS have demonstrated good diagnostic ability to identify nodules for FNA, their performance is poorer in nodules with a diameter greater than 20 mm. In one study, ACR TI-RADS demonstrated a slightly higher specificity, suggesting its potential utility in reducing unnecessary biopsies in this subset of patients (17). In a previous study from our group focusing on nodules greater than 20 mm, the combination of EU-TIRADS with RTE enhanced diagnostic accuracy, particularly for low-risk nodules (EU-TIRADS 3). The integration of these tools may help reduce unnecessary FNA and surgical interventions (18).

In nodules subjected to FNA, elastography was statistically significant in the overall prediction of malignancy, as determined using a mixed predictive model incorporating clinical and US parameters. However, its sensitivity (76.4%) and specificity (87.2%) were insufficient to support its use as a standalone diagnostic tool for malignancy risk assessment. Using an ER threshold of greater than 1.13, a nodule was approximately 6 times more likely to be malignant. This threshold was derived from the overall cohort and applies to all nodules regardless of their grayscale or ACR TI-RADS category, representing a global estimate of elastographic performance. Considering the disease prevalence (9.89%) and the estimated diagnostic cost, an alternative threshold (>1.94) was proposed that provided higher specificity (97.6%) but lower sensitivity (49.1%), suggesting its potential utility in confirming malignancy in selected cases.

Notably, elastography demonstrated considerable diagnostic value when employed as an adjunct to stratify suspicious nodules within each ACR TI-RADS category. The proposed ER thresholds for each category (ACR TI-RADS 3: >1.60, ACR TI-RADS 4: >1.37, ACR TI-RADS 5: >0.95) could have avoided a substantial number of FNAs (ACR TI-RADS 3:159/160 [96.9%], ACR TI-RADS 4:276/312 [88.5%], ACR TI-RADS 5:23/29 [79.3%]).

A noteworthy finding was that the additive value of elastography was most pronounced in nodules with low malignant potential (for ACR TI-RADS 3, the sensitivity was 100%). This is clinically relevant because most “unnecessary” aspirations are performed in this category (19, 20).

In contrast, the sensitivity of elastography was 80.6% for ACR TI-RADS 4 nodules and 70% for ACR TI-RADS 5 nodules. Although the ER cutoff for TI-RADS 4 exceeded that for TI-RADS 5, this likely reflects the smaller number of TI-RADS 5 nodules (n = 49) and the reduced variability of stiffness values within this high-risk group, resulting in a less stable ROC-derived threshold (AUC 0.812; 95% CI, 0.633-1.812). Therefore, this cutoff should be interpreted with caution and validated in larger cohorts.

To improve sensitivity while reducing unnecessary procedures, applying a lower ER threshold of less than 0.44 in the ACR TI-RADS 4 category would have avoided 71 of 312 (22.8%) FNAs, and a threshold of less than 0.54 in the ACR TI-RADS 5 category would have avoided 11 of 29 (38%) FNAs without missing any malignant cases.

The integration of elastography into thyroid nodule RSS aligns with the broader health-care goal of reducing unnecessary interventions and optimizing diagnostic precision. Our findings confirm that elastography enhances the diagnostic performance of ACR TI-RADS, supporting more accurate decision-making, particularly for intermediate-risk nodules. This is consistent with the results of a prior study by Li et al (21), in which the addition of elastography improved the predictive accuracy of TIRADS 4 nodules among high-risk populations confirmed by thyroidectomy. Several studies (22-24) have demonstrated the ability of elastography to reliably differentiate malignant from benign lesions, with thresholds for the ER ranging from greater than 2.0 to greater than 2.3 and sensitivities up to 95.2%. Despite these findings, elastography remains underused in clinical practice. Nonetheless, elastography has been recognized as an independent prognostic factor, and the World Federation for Ultrasound in Medicine and Biology guidelines acknowledge tissue stiffness as a relevant marker of malignancy (25). In parallel, the shift toward the active surveillance of low-risk thyroid cancers further underscores the need for accurate, noninvasive diagnostic tools (26).

Most studies estimated ER by comparing the strain in the nodule to the adjacent thyroid parenchyma at similar depths to minimize variability due to tissue anisotropy and probe pressure (27, 28). However, fibrosis or lymphocytic infiltration may unpredictably affect tissue deformability (29). In our study, to address this limitation and standardize the measurements, we used the sternocleidomastoid muscle as a reference structure, despite the different imaging depths. This alternative approach may offer greater reproducibility than the conventional approach. However, the use of the sternocleidomastoid muscle as the reference tissue in SR calculations may introduce bias, as muscle stiffness can vary with age and sex. In our cohort, no statistically significant differences in muscle stiffness were observed according to sex or age (data not shown); nevertheless, this factor should be acknowledged as a potential source of variability that may limit reproducibility across different populations.

Furthermore, although elastography is considered less operator-dependent than grayscale US, image quality and interpretation can still benefit from structured training (30). Overall, our data support the incorporation of elastography thresholds into future revisions of the ACR TI-RADS. Nonetheless, further prospective validation in diverse settings is essential to ensure the generalizability and clinical applicability of the proposed thresholds.

Our study also assessed the influence of additional clinical variables (BMI, T4 supplementation, and history of autoimmune thyroiditis) on the rate of malignant cytology in the ACR TI-RADS categories. Several studies have suggested a potential association between an increased BMI and a higher risk of thyroid cancer, particularly in women (31, 32). This association could be mediated by hormonal alterations, obesity-related inflammation, and insulin resistance, which are more prevalent in individuals with an elevated BMI (33). Moreover, Di Filippo et al (34) reported that higher BMI was associated with more aggressive histopathological subtypes of differentiated thyroid carcinoma, while Wang et al (35) observed that overweight and obese patients had increased odds of presenting with advanced Tumor-Node-Metastasis (TNM) stages. Interestingly, in our study, a negative association was found between BMI and the presence of malignancy in nodules classified as intermediate-risk (ACR TI-RADS 4). This unexpected finding aligns with the results of the NIH-AARP Diet and Health Study cohort analyzed by Kitahara et al (36), which showed that although a higher BMI was generally associated with an increased thyroid cancer risk, the strength of the association varied by histological subtype and was not significant for certain subtypes, such as follicular carcinoma. Furthermore, the same group of authors (37) highlighted the possibility of detection bias contributing to the observed link between obesity and thyroid cancer, as individuals with a higher BMI are more likely to undergo medical imaging, leading to the incidental detection of thyroid nodules or malignancies. Given the limited number of malignant cases in this category in our cohort, larger studies are required to confirm this finding.

Whether chronic lymphocytic thyroiditis represents a risk factor for differentiated thyroid carcinoma remains under investigation (38-40). In our cohort, autoimmune thyroiditis was present in 27.9% and hypothyroidism in 28.8% of patients. These conditions were not independently associated with nodule malignancy in our analysis; their prevalence underscores the frequent coexistence of autoimmune thyroid disease among patients evaluated for thyroid nodules. The diagnosis of autoimmune thyroiditis in our cohort was based on a history of elevated antithyroid antibody titers. Since antibody levels may decline over time and become negative in long-standing diseases, some cases could have been misclassified as nonautoimmune hypothyroidism.

Regarding the findings from CDUS, vascular patterns used to assess thyroid nodules have been studied and found to differentiate between benign and malignant lesions. In our study, patients with nodules demonstrating peripheral vascularity were less likely to have malignancies than those with mixed or central vascularity. Although hypervascularity is not a specific sign of malignancy, several studies have supported this observation (41-43), as hypervascularity has been observed in 69% to 74% of malignant cases (44). Benign nodules are typically characterized by peripheral vascularity, which can be observed in up to 22% of carcinomas (44). Intranodular (central) vascularity is commonly observed in malignant nodules but lacks specificity. “Chaotic” or disorganized vascular patterns are considered more specific for malignancy, albeit with very low sensitivity (45). Central vascularity is the most specific US feature of nodules with indeterminate cytology, with a specificity of 96% and positive likelihood ratio of 2.13 (46). The absence of vascularity in solid thyroid nodules does not indicate benignity or malignancy. Some studies suggest that a lack of blood flow may be more indicative of benignity, particularly in colloid nodules or those with fibrotic features. However, the diagnostic significance of avascularity in solid, noncystic nodules remains unclear. In our study, the absence of vascularity in thyroid nodules (excluding cystic or colloid nodules, as categorization was based on the ACR TI-RADS system) was a potentially adverse indicator of malignancy. Nevertheless, the number of nodules exhibiting this pattern was small, and the clinical significance of this observation needs to be evaluated in larger studies. In our multivariate analysis, the absence of vascularity emerged as the most statistically significant independent predictor of malignancy. This finding is consistent with previous sonographic-pathologic studies demonstrating that most malignant nodules, particularly papillary thyroid carcinomas, lack intranodular vascularity on CD imaging (46). The absence of vascularization may therefore represent a simple and reproducible Doppler feature to aid risk stratification, although external validation in larger cohorts is required.

These findings are consistent with the underlying histopathology of the thyroid lesions. Follicular-pattern tumors are usually surrounded by a fibrous capsule rich in blood vessels, which sonographically appear as well-defined nodules with a halo and peripheral vascularization, typically falling within the TR3 to TR4 categories. In contrast, papillary thyroid carcinomas generally lack a capsule and therefore often exhibit either no vascularization or central vascularization, explaining why such patterns were observed primarily in TR5 nodules and never in TR3 (47, 48). Among the vascularization patterns, the absence of vascularization showed the strongest association with malignancy in our series, while the mixed pattern was less consistently associated with malignancy and was likely less reproducible across operators, US systems, and Doppler settings.

An important limitation of applying adjunctive parameters to ACR TI-RADS is the lack of specific guidance on how to incorporate factors such as vascular flow patterns and patient demographics into risk stratification. Although these features may influence clinical decision-making, their integration remains undefined in the current protocols. For instance, it is unclear whether a small TI-RADS 3 nodule with suspicious Doppler flow should prompt a biopsy despite not meeting the size threshold. The present study was not designed to define a clear algorithm for integrating such parameters; however, it highlights their potential relevance and underscores the need for further research to determine how they may enhance the performance of existing classification systems.

Despite these limitations, our results are consistent with recent evidence supporting the diagnostic role of elastography in thyroid nodules. A large randomized controlled trial (49) demonstrated the added value of US elastography in malignancy prediction, while a systematic review and meta-analysis (50) confirmed its utility across different elastography techniques. These findings strengthen the rationale for incorporating elastography into thyroid risk stratification systems, although reproducibility issues related to operator experience, equipment differences, and acquisition protocols remain considerable challenges for clinical implementation.

Our study adds to the existing literature by addressing 2 specific knowledge gaps. First, although the diagnostic value of elastography has been repeatedly demonstrated, its integration with structured RSSs, such as the ACR TI-RADS, has not been fully explored. We demonstrated that RTE may enhance TI-RADS–based risk stratification, particularly in low-risk (TR3) nodules, where unnecessary FNAs could be avoided. Second, we showed that the absence of vascularity was the strongest independent predictor of malignancy in our multivariate analysis. This Doppler feature, which has rarely been emphasized in previous TI-RADS–focused studies, may provide additional value for clinical decision-making. Together, these findings highlight the potential of combining TI-RADS with elastography and Doppler imaging to refine the management of thyroid nodules.

One methodological limitation of this study is the inclusion of only the largest thyroid nodule per patient for analysis, which was implemented to ensure statistical independence and to avoid clustering bias. However, this approach may have excluded smaller nodules with higher TI-RADS scores or greater malignant potential, potentially underestimating the true prevalence of high-risk nodules in the population. Although all nodules meeting the biopsy criteria were sampled in clinical practice, their exclusion from the final dataset may have influenced the distribution of cytological outcomes and risk stratification. Our study was also limited because cytological results were available only for nodules that met the criteria for FNA according to the ACR TI-RADS recommendations. Consequently, the diagnostic performance of elastography was evaluated only in patients with FNA results. Ideally, cytological data from all examined nodules would have been valuable for a comprehensive comparison of the sensitivity and specificity of elastography relative to the ACR TI-RADS stratification. Despite this limitation, we have shown that integrating elastography and selected clinical variables into the RSS can improve diagnostic efficiency and support more cost-effective, individualized patient management.

Another limitation of our study is that all examinations were performed by a single, highly experienced operator using a single US system. While this design ensured homogeneity and minimized intraobserver variability, it limited the generalizability of our results to other operators and US platforms. Both elastography and Doppler evaluations are known to be affected by interobserver variability and technical differences between equipment. Therefore, our findings require external validation in multicenter settings with different operators and US systems to confirm their applicability in broader clinical practices. Another limitation is the particular characteristics of our cohort. The rate of benign cytology (86%) was higher, and the proportion of indeterminate cytology (Bethesda III-IV) was lower than that usually reported, while the malignancy rates for Bethesda IV to V were higher. The prevalence of malignancy among TR3 nodules was also lower (2.5% vs ∼5% in other series), limiting the reliability of further stratification with elastography in this group of patients. Furthermore, the relatively small number of TI-RADS 5 nodules (n = 49) limited the precision of the ROC-derived ER threshold in this category, and the corresponding cutoff should therefore be interpreted with caution and validated in larger datasets. In addition, no true follicular thyroid carcinomas were observed, as the series was enriched with papillary thyroid carcinomas, including follicular variants, while no medullary or oncocytic (Hürthle cell) carcinomas were represented in our cohort. These histological variants may differ in vascularization patterns or elastographic characteristics, which could potentially influence imaging-based risk assessment. Therefore, our findings specifically reflect the diagnostic performance of elastography in differentiating benign nodules from papillary thyroid carcinoma, rather than thyroid cancer in general. These features may have reduced the generalizability of our results. The cutoff values derived from the ROC analysis were not validated in an independent cohort. Therefore, they should be interpreted with caution and considered hypothesis-generating, requiring external validation in future studies, before clinical implementation.

## Data Availability

The data supporting the findings of this study are available in the supplementary materials deposited in Figshare at DOI: 10.6084/m9.figshare.30535406.

## Abbreviations

ACR TI-RADS: American College of Radiology Thyroid Imaging Reporting and Data System
AUC: area under the curve
BMI: body mass index
CDUS: color Doppler US
ER: elastography ratio
FN: false negative
FNA: fine-needle aspiration
FP: false positive
ROC: receiver operating characteristic
RSS: risk stratification systems
RTE: real-time elastography
SE: strain elastography
SR: strain ratio
T_4_: thyroxine
TN: true negative
TP: true positive
US: ultrasonography
